# Open and closed structures of L-arginine oxidase by cryo-electron microscopy and X-ray crystallography

**DOI:** 10.1093/jb/mvae070

**Published:** 2024-10-18

**Authors:** Hiroki Yamaguchi, Kazutoshi Takahashi, Nobutaka Numoto, Hiroshi Suzuki, Moemi Tatsumi, Akiko Kamegawa, Kouki Nishikawa, Yasuhisa Asano, Toshimi Mizukoshi, Hiroshi Miyano, Yoshinori Fujiyoshi, Masayuki Sugiki

**Affiliations:** Research Institute for Bioscience Products & Fine Chemicals, Ajinomoto Co. Inc., 1-1 Suzuki-cho, Kawasaki-ku, Kawasaki 210-8681, Japan; Advanced Research Institute, Tokyo Medical and Dental University, 1-5-45 Yushima, Bunkyo-ku, Tokyo 113-8501, Japan; Research Institute for Bioscience Products & Fine Chemicals, Ajinomoto Co. Inc., 1-1 Suzuki-cho, Kawasaki-ku, Kawasaki 210-8681, Japan; Medical Research Institute, Tokyo Medical and Dental University, 1-5-45 Yushima, Bunkyo-ku, Tokyo 113-8501, Japan; Advanced Research Institute, Tokyo Medical and Dental University, 1-5-45 Yushima, Bunkyo-ku, Tokyo 113-8501, Japan; Research Institute for Bioscience Products & Fine Chemicals, Ajinomoto Co. Inc., 1-1 Suzuki-cho, Kawasaki-ku, Kawasaki 210-8681, Japan; Advanced Research Institute, Tokyo Medical and Dental University, 1-5-45 Yushima, Bunkyo-ku, Tokyo 113-8501, Japan; CeSPIA Inc., 2-1-1 Otemachi, Chiyoda-ku, Tokyo 100-0004, Japan; Joint Research Course for Advanced Biomolecular Characterization, Faculty of Agriculture, Tokyo University of Agriculture and Technology, 3-5-8 Saiwai-cho, Fuchu, Tokyo 183-8509, Japan; CeSPIA Inc., 2-1-1 Otemachi, Chiyoda-ku, Tokyo 100-0004, Japan; Joint Research Course for Advanced Biomolecular Characterization, Faculty of Agriculture, Tokyo University of Agriculture and Technology, 3-5-8 Saiwai-cho, Fuchu, Tokyo 183-8509, Japan; Biotechnology Research Center and Department of Biotechnology, Toyama Prefectural University, 5180 Kurokawa, Imizu, Toyama 939-0398, Japan; Research Institute for Bioscience Products & Fine Chemicals, Ajinomoto Co. Inc., 1-1 Suzuki-cho, Kawasaki-ku, Kawasaki 210-8681, Japan; Research Institute for Bioscience Products & Fine Chemicals, Ajinomoto Co. Inc., 1-1 Suzuki-cho, Kawasaki-ku, Kawasaki 210-8681, Japan; Advanced Research Institute, Tokyo Medical and Dental University, 1-5-45 Yushima, Bunkyo-ku, Tokyo 113-8501, Japan; CeSPIA Inc., 2-1-1 Otemachi, Chiyoda-ku, Tokyo 100-0004, Japan; Joint Research Course for Advanced Biomolecular Characterization, Faculty of Agriculture, Tokyo University of Agriculture and Technology, 3-5-8 Saiwai-cho, Fuchu, Tokyo 183-8509, Japan; Research Institute for Bioscience Products & Fine Chemicals, Ajinomoto Co. Inc., 1-1 Suzuki-cho, Kawasaki-ku, Kawasaki 210-8681, Japan

**Keywords:** amino acid oxidase, cryo-EM, L-arginine oxidase, single particle analysis, X-ray crystallography

## Abstract

L-arginine oxidase (AROD, EC 1.4.3.25) is an oxidoreductase that catalyses the deamination of L-arginine, with flavin adenine dinucleotide (FAD) as a cofactor. Recently identified AROD from *Pseudomonas* sp. TPU 7192 (PT-AROD) demonstrates high selectivity for L-arginine. This enzyme is useful for accurate assays of L-arginine in biological samples. The structural characteristics of the FAD-dependent AROD, however, remain unknown. Here, we report the structure of PT-AROD at a resolution of 2.3 Å by cryo-electron microscopy. PT-AROD adopts an octameric structure with D4 symmetry, which is consistent with its molecular weight in solution, estimated by mass photometry. Comparative analysis of this structure with that determined using X-ray crystallography reveals open and closed forms of the lid-like loop at the entrance to the substrate pocket. Furthermore, mutation of Glu493, located at the substrate binding site, diminishes substrate selectivity, suggesting that this residue contributes significantly to the high selectivity of PT-AROD.

## Graphical Abstract

**Graphical Abstract:**
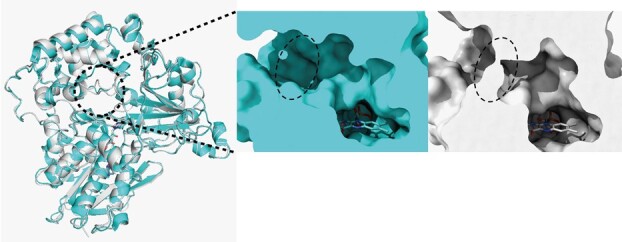

## Abbreviations

AncLLysO: ancestral lysine oxidase
AROD: L-arginine oxidase
cryo-EM: cryo-electron microscopy
CTF: contrast transfer function
FAD: flavin adenine dinucleotide
FSC: Fourier shell correlation
HEPES: 4-(2-hydroxyethyl)-1-piperazineethanesulfonic acid
LAAO: l-amino acid oxidase
PT-AROD: AROD from *Pseudomonas* sp. TPU 7192
RMSD: root-mean-square deviation
SEC: size exclusion chromatography
TOOS: N-ethyl-N-(2-hydroxy-3-sulfopropyl)-3-methylaniline sodium salt

Amino acids are essential for life and play crucial roles in various metabolic pathways, including protein synthesis and nitrogen metabolism. Their importance has stimulated extensive research in fields as diverse as biochemistry and medicine. Amino acids are characterized using instrumental and enzymatic methods. Instrumental methods include high-performance liquid chromatography and electrospray ionization tandem mass spectrometry *(**1*–*3**)*, whilst enzymatic methods, which are simple and accurate, use enzymes with high specificity for a single type of amino acid to obtain reaction products that are typically detected using chromogenic or ultraviolet methods *(**4**,**5**)*. Oxidase and dehydrogenase are enzymes applied to react with amino acids *(**6**)*. Our study specifically focuses on oxidase, namely L-amino acid oxidase (LAAO), which is the most commonly used enzyme in these assays.

LAAOs are amino acid-metabolizing enzymes that catalyse the oxidation of the main-chain amino group of L-amino acids to produce α-keto acids and ammonia *(**7**)*. LAAOs are categorized into two groups: flavin adenine dinucleotide (FAD)-dependent *(**8*–*10**)* and FAD-independent enzymes *(**11*–*13**)*. LAAOs facilitate the conversion of L-amino acids into D-amino acids, which serve as precursors of pharmaceutical compounds, thereby playing a crucial role in biotechnology *(**7**)*. Enzymes with broad substrate selectivity have been identified in diverse species, including bacteria *(**14**)*, fungi *(**15**)* and venomous snakes *(**16**)*. Enzymes with high substrate specificity, such as L-arginine oxidase (AROD) *(**17**)*, L-glutamate oxidase *(**9**,**18**)* and L-tryptophan oxidase *(**5**)*, which are named according to their distinct specificity, have also been reported. These application of these enzymes in assays is useful for determining the concentration of L-amino acids in various samples.

ARODs that catalyse the deamination of L-arginine have been derived from *Pseudomonas putida* P2 *(**19**,**20**)* and cyanobacterium *Synechococcus elongatus (**21**)*. In addition, ancestral ARODs have been designed using computational methods *(**22**)*. Recently, Matsui et al. identified FAD-dependent AROD (EC 1.4.3.25, GenBank: HW613270.1) from *Pseudomonas* sp. TPU 7192 (PT-AROD) by screening microorganisms *(**17**)*. They demonstrated that PT-AROD selectively recognizes L-arginine amongst 20 L-amino acids and is thus a useful enzyme for assays of L-arginine. Previous studies have shown that the sequence of PT-AROD is similar to those of the putative monoamine oxidase of *P. putida* (accession WP_009408839) and the putative amine oxidoreductase of *Pseudomonas japonica* (accession WP_042125990) but not to those of LAAOs *(**17**)*. Few studies, however, have examined the function and structure of FAD-dependent ARODs, and experimental structures have not been reported. At the commencement of our study, we found that amongst the proteins with known structures in the Protein Data Bank (PDB), the protein with the highest degree of sequence homology with PT-AROD shared ~32% of its sequence. Therefore, elucidating the structure of this enzyme will not only deepen our understanding of ARODs but also provide new insights into LAAOs. Here, we present the high-resolution structure of the AROD derived from *Pseudomonas* sp. TPU 7192, which was characterized using X-ray crystallography and single-particle cryo-electron microscopy (cryo-EM). Moreover, we designed mutants using structural information to investigate the substrate specificity of PT-AROD.

## Material and Methods

### Protein expression and purification

The pET-15b plasmid in which PT-AROD was cloned was provided by Dr. Asano’s laboratory, at Toyama Prefectural University *(**17**)*. PT-AROD was fused with the sequence MGSSHHHHHHSSGLVPRGSH at the N-terminus, and the sequences GSS and SSGLVPRGSH were removed by inverse-polymerase chain reaction.

The primer sequences were as follows: 5′- TACCATGCATCACCATCACCATCACATGAG-3′ and 5’-GGTGATGGTGATGCATGGTATATCTCCTTC-3′ for the deletion of GSS, and 5′- ATGAGCCAGACCCAGCCATTGGATGTCGCCATC-3′ and 5′- GTGATGATGATGATGATGGCTGCTGCCCATGG-3′ for the deletion of SSGLVPRGSH. The plasmid was introduced into *Escherichia coli* BL21 (DE3), and colonies were inoculated in LB medium containing 100 μg/ml ampicillin at 37°C. His-tag fusion proteins were expressed by induction using 1 mM isopropyl-β-D-thiogalactopyranoside when the OD_600_ reached 0.6. The temperature was decreased to 16°C, followed by incubation for 16 h. Harvested cells were washed with saline, then re-suspended in lysis buffer containing 50 mM HEPES (pH 7.5), 500 mM NaCl, 50 mM imidazole and 0.2 μM FAD, and disrupted with an ultrasonic disintegrator (201 M, KUBOTA, Tokyo, Japan). The supernatant was added to a HisTrap FF crude column (Cytiva, Marlborough, MA, USA), and bound proteins were eluted with a gradient of 50–500 mM imidazole. Peak fractions were pooled, diluted 10-fold with a buffer containing 20 mM HEPES (pH 8.0) and 0.2 μM FAD and were applied to a Resource Q column (Cytiva) and eluted with a gradient of 0–1,000 mM NaCl. Finally, the protein solution was applied to a Superdex 200 Increase 10/300 GL column (Cytiva) with SEC buffer containing 20 mM HEPES (pH 8.0) and 50 mM NaCl. All purification procedures were performed at 4°C.

### Cryo-EM data acquisition and image processing

The protein sample was concentrated to 2.5 mg/ml using an AmiconUltra filter (30 kDa cut-off, Merck Millipore, Burlington, MA, USA), and 3.5 μl aliquots were loaded onto glow-discharged Quantifoil holey carbon grids (R1.2/1.3 Au, 300 mesh), blotted for 4.0 s at 4°C, and plunge-frozen in liquid ethane using a Vitrobot Mark IV (Thermo Fisher Scientific, Waltham, MA, USA). Images were acquired at 300 kV on a JEM-Z320FHC microscope (JEOL, Tokyo, Japan), which was cooled by liquid nitrogen and equipped with a cold field-emission gun and an in-column-type energy filter. All images were recorded on a K2 Summit direct electron detector (Gatan) operated in the electron counting mode using SerialEM *(**23**)*. The calibrated pixel size on the specimen was 0.96 Å, and 8.0-s exposures were dose-fractionated into 40 frames with an electron flux of 8 e^−^/pix/s, resulting in an accumulated dose of 69.6 e^−^/Å^2^.

**Table 1:** Cryo-EM data collection, refinement and validation statistics

		AROD (EMDB: EMD-36635) (PDB:8JT7)
Microscope		JEM-Z320FHC
Detector		K2 Summit
Magnification		50,000
Voltage (kV)		300
Exposure time (s)		8.0
Electron exposure (e^−^/Å^2^)		69.6
Defocus range (μm)		1.0–2.0
Pixel size (Å)		0.96
Symmetry imposed		D4
Initial particle images (*n*)		3,387,866
Final particle images (*n*)		254,111
Map resolution (Å)		2.34
FSC threshold		0.143
**Refinement**		
Map CC (mask)		0.7298
Map CC (volume)		0.7265
Map sharpening B factor (Å^2^)		−52.8
Model composition		
	Protein residues	4,632
	Protein atoms	36,864
	FAD molecules	8
	Water	232
B factors (Å^2^)		
	Protein	80.97
	FAD	63.18
RMSD		
	Bond lengths (Å)	0.01
	Bond angles (°)	1.507
Validation		
	Molprobity score	1.59
	Clashscore	4.08
	Poor rotamers (%)	3.1
Ramachandran plot		
	Favoured (%)	97.91
	Allowed (%)	2.09
	Disallowed (%)	0

The data were processed using RELION-4.0 *(**24**)*. The beam-induced motion of 5,035 image stacks was corrected using MotionCor2 *(**25**)*. The contrast transfer function (CTF) parameters of each micrograph were estimated using CTFFIND4 *(**26**)*. To obtain references for particle picking, 500 ‘bright’ (i.e. large file size) movies were selected and subjected to LoG-based auto-picking in RELION. The particles were extracted with down-sampling to a pixel size of 1.92 Å and subjected to 2D classification. Representative class averages with fine structural detail were selected for template-based auto-picking. In total, 3,387,866 particles were extracted with down-sampling to a pixel size of 2.88 Å and subjected to 2D classification. The obtained 2D class averages were predominantly ‘side views’, with ~5% of particles being top views. Because an interpretable 3D model could not be obtained *de novo* by 3D model generation using all the particles, 2D classes that did not show the side view were intentionally selected and the corresponding 158,415 particles yielded a good initial map with a D4 symmetric feature. Multiple rounds of 3D classification using gradually increased numbers of particles corresponding to the side-view class were performed with C1 symmetry, and 254,111 particles were selected and re-extracted to a pixel size of 0.96 Å. The particles were subjected to 3D auto-refinement with D4 symmetry followed by CTF refinement and Bayesian polishing. The final round of 3D auto-refinement (D4 symmetry) and post-processing yielded a map with an overall resolution of 2.34 Å according to the Fourier shell correlation (FSC) criterion of 0.143 *(**27**)*.

### Model building and refinement

Model building using Coot *(**28**)* and real-space refinement in PHENIX *(**29**)* were iterated for several cycles. Refinement in REFMAC5 *(**30**)* was performed in the Servalcat pipeline *(**31**)*. The final model was visually inspected for general fit to the map, and the geometry was further evaluated using MolProbity *(**32**)*. The final refinement statistics are summarized in Table 1. Molecular graphics were prepared using UCSF ChimeraX *(**33**)*, UCSF Chimera *(**34**)* and PyMOL version 2.5.4.

### Crystallization

Crystals of PT-AROD were grown at 20°C from a 1:1 mixture of the protein solution (10 mg/ml) with 20% w/v polyethylene glycol 3350 and 0.2 M potassium citrate tribasic monohydrate (pH 8.3) (Hampton Research, Aliso Viejo, CA, USA). Crystals of PT-AROD were cryoprotected by soaking in a solution containing 20% w/v polyethylene glycol 3350, 0.2 M potassium citrate tribasic monohydrate (pH 8.3) and 15% glycerol and then flash-frozen in liquid nitrogen.

### X-ray data collection, phase determination and refinement

Diffraction patterns of PT-AROD were collected on beamline PF-BL-5A at the Photon Factory (Tsukuba, Japan). Data were collected at 100 K. Crystals of PT-AROD were diffracted to 3.4 Å. The collected data were processed with XDS, and an initial phase was obtained using MOLREP *(**35**)*. For model building, we carried out structure refinement using CCP4 REFMAC *(**36**)* and manual rebuilding with Coot *(**28**)*. A tetrameric molecule was contained in the asymmetric unit. The final structure was deposited in the PDB as 8T8A. Table 2 summarizes data collection and refinement statistics.

**Table 2:** Data collection and refinement statistics

	AROD (PDB: 8T8A)
Data reduction	
Space group	*C* 2 2 2_1_
Cell constants	*a* = 168.27 Å, *b* = 200.22 Å, *c* = 168.54 Å, *α* = 90.00, *β* = 90.00, *γ* = 90.00
Resolution (Å)	49.23–3.40 (3.47–3.40)
% Data completeness	100.0 (100.0)
*I*/σ(*I*)	4.6 (2.1)
Wilson *B*-factor (Å^2^)	85.5
Refinement	
Total reflections	37,466
*R*_work_, *R*_free_	0.212, 0.313
Total number of atoms	17,774
B factors (Å^2^)	
Protein	93.18
FAD	93.12
RMSD	
Bond lengths (Å)	0.004
Bond angles (°)	1.421
Ramachandran plot	
Favoured (%)	82.86
Allowed (%)	14.73
Disallowed (%)	1.89

**Fig. 1:**
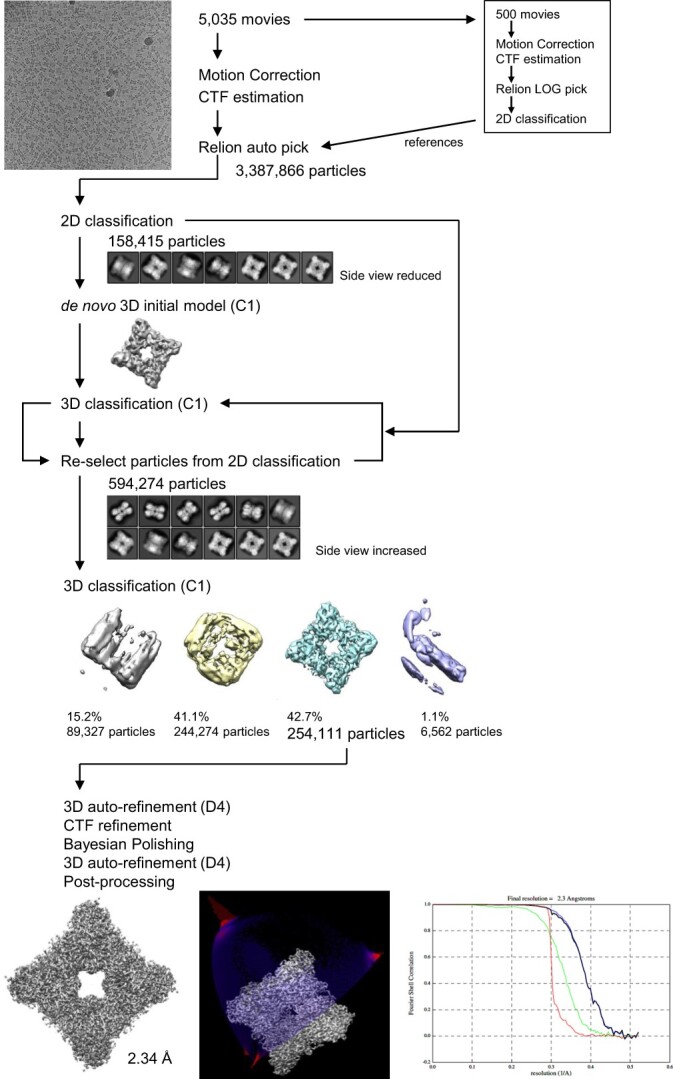
**Cryo-EM analysis flow chart of the cryo-EM data processing for L-arginine oxidase from *Pseudomonas* sp. TPU 7192.** Processing workflow for PT-AROD structure determination by single-particle analysis. Gold-standard FSC curves (right bottom) for PT-AROD are displayed after applying no mask (green), a mask (blue) or phase-randomized mask (red). The corrected FSC curve is shown in black. All images in this figure were created in UCSF Chimera.

**Fig. 2:**
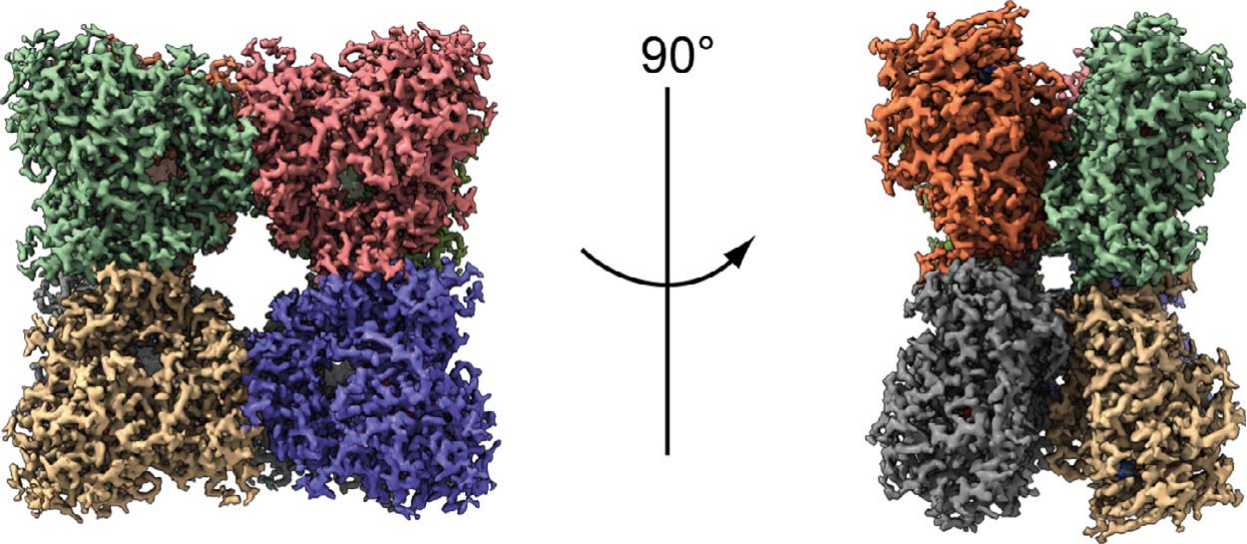
**Cryo-EM map of L-arginine oxidase from *Pseudomonas* sp. TPU 7192.** The density map of PT-AROD is shown at a contour level 0.05. Density maps applicable to each monomer are shown in different colours. All images in this figure were created in UCSF ChimeraX.

### Mass photometry

Mass photometry (OneMP, Refeyn, Oxford, UK) measurements were performed on a glass coverslip for 60 s. Each measurement was repeated three times. The samples were diluted to 0.1 mg/ml in SEC buffer immediately prior to the measurements. The recorded videos were analysed using DiscoverMP (Refeyn, version 2.2.0) to quantify protein-binding events. The molecular weight was obtained by contrast comparison with known mass standard calibrants *(**37**)*.

### Enzymatic assay

PT-AROD activity was quantified using a colourimetric assay. The reaction mixture, containing 100 mM Tris–HCl (pH 7.0), 10 mM substrate (L-arginine or L-lysine), 15 U/ml peroxidase, 1 mM 4-aminoantipyrine, 3 mM N-ethyl-N-(2-hydroxy-3-sulfopropyl)-3-methylaniline (TOOS, Dojindo, Kumamoto, Japan) and 1 μg/ml PT-AROD solution, was diluted to 1 μg/ml in a final volume of 200 μl. The absorbance at 555 nm was measured at 37°C using a microplate reader (Varioskan LUX, ThermoFisher). Kinetics were measured using L-arginine and L-lysine as substrates at final concentrations ranging from 0 and 2.5 mM, and 0 and 10 mM, respectively. The kinetic parameters were determined through non-linear regression analysis using GraphPad Prism 6.07 (GraphPad Software Inc., San Diego, CA, USA). The substrate specificity of PT-AROD for each amino acid was analysed by measuring the reaction at 37°C for 5 min using 10 mM of each amino acid. To ensure storage stability, the PT-AROD solution was kept at 4°C under light-shielded conditions prior to use. Changes in the absorbance at 605 nm over time were measured using a clinical chemistry analyser (TBA-120FR, Canon Medical Systems, Ohtawara, Japan).

**Fig. 3:**
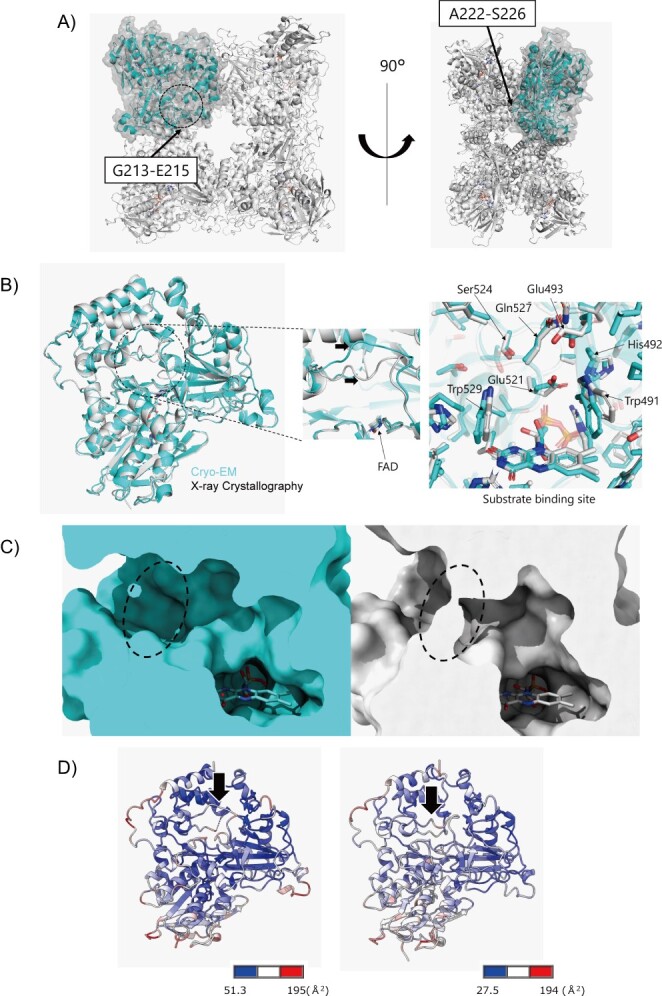
**Structural comparison between Cryo-EM and X-ray crystallography.** (A) PT-AROD A chain (cyan) and B–H chain (white) from cryo-EM are shown as a cartoon model. Three residues (G213–E215) are disordered, and the ATEYS (Ala-Thr-Glu-Tyr-Ser) loop (A222–S226) differs. (B) Superimposed cryo-EM (cyan) and X-ray crystallography (white) structures. Dotted circle shows the ATEYS loop. (C) Side view of the substrate binding pocket. The cryo-EM structure is in an open conformation (left), whilst the X-ray crystallography structure is in a closed conformation (right). PT-AROD is shown in surface representation, and FAD is shown as a stick model. (D) The cryo-EM (left) and X-ray crystallography (right) structures of PT-AROD are coloured according to the temperature factor. Black arrows indicate the ATEYS loops.

### Site-directed mutagenesis of PT-AROD

Mutants of PT-AROD were prepared by site-directed mutagenesis using KAPA HiFi DNA polymerase (Nippon Genetics, Tokyo, Japan). The primer sequences were as follows: TGCTGGTGGAATCGGCCTTCAAGCTG-3′ and 5’-CAGCTTGAAGGCCGATTCCACCAGCACCGAGTC-3′ for Q350E, and 5’-GCGGCGGCTGGCACGCATGGAAGGCCAACTAC-3′ and 5’-GTAGTTGGCCTTCCATGCGTGCCAGCCGCCGC-3′ for E493A. The plasmids were introduced into *E. coli* XL10-Gold for plasmid purification. Protein expression and purification followed the same procedure as for the wild type. The activity was quantified using the enzymatic assay described above.

## Results and Discussion

### Overall structure of PT-AROD determined by single particle analysis

PT-AROD was expressed in *E. coli* and subsequently purified using a His-tag, with further purification using anion-exchange and size-exclusion chromatography. We obtained a cryo-EM map of PT-AROD in complex with FAD at a resolution of 2.3 Å (Fig. 1), which was suitable for *de novo* model building (Table 1). PT-AROD has an octameric structure with two layers of tetramers and one molecule of FAD bound to each monomer (Fig. 2). This structure was consistent with that obtained from mass spectrometry in a previous study *(**17**)* and in this study (Supplementary Fig. S1). Therefore, PT-AROD is a homooctamer, whilst most LAAOs are known to be dimers *(**7**)*. Interestingly, disulphide bonds between Cys390 and Cys579 in neighbouring subunits were observed at the interface of the octamer (Supplementary Fig. S2). In general, disulphide bonds contribute to protein stability *(**38**,**39**)*. Indeed, Matsui et al. reported that PT-AROD retains >95% of its activity after treatment at 60°C for 30 min in a pH 7.0 environment. In addition, we confirmed that purified PT-AROD retained its enzymatic activity after storage for 1.5 years at 4°C under light-shielded conditions (Supplementary Fig. S3). This high stability of PT-AROD is likely due to in the formation of a stable complex via disulphide bonds.

**Fig. 4:**
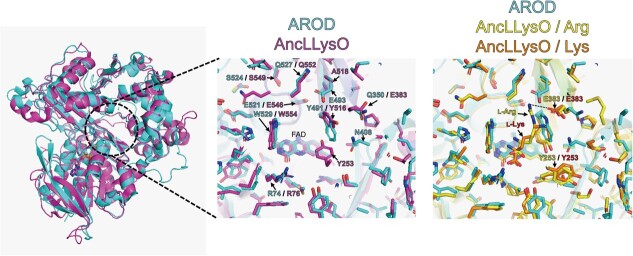
**Substrate binding site of L-arginine oxidase and L-lysine oxidase.** (Left) Superposition of FAD-bound monomeric subunits of PT-AROD (cyan, cryo-EM) and AncLLysO (magenta, PDB: 7EIH). (Middle) Close-up view of the residues around the putative substrate site, shown as a stick model. (Right) Comparison of the substrate sites of PT-AROD/FAD (cyan, cryo-EM), AncLLysO/FAD/L-arginine complexes (yellow, PDB: 7EIJ) and AncLLysO/FAD/L-lysine (orange, PDB: 7EII). Dotted line indicates a hydrogen bond between Glu383 and L-arginine side chains.

### Comparison with structure determined using X-ray crystallography

We also determined the structure of PT-AROD using X-ray crystallography at a resolution of 3.4 Å. Molecular replacement was performed using the structure in AlphaFold2 *(**40**)* as a template. Under our conditions, four molecules were present in the asymmetric unit. By generating a symmetry mate, we replicated the same octameric assembly of PT-AROD obtained by cryo-EM analysis (Supplementary Fig. S4). Comparative analysis of the X-ray crystallography and cryo-EM structures revealed that the root-mean-square deviation (RMSD) of the Cα atoms between the monomers and generated octamers was 0.644 and 1.021 Å, respectively, and no significant differences were observed in the overall structures. In the protomer, two slight conformational differences were observed near the substrate binding pocket. First, loop 213–215 located at the molecular interface between neighbouring subunits was disordered in the cryo-EM structure (Fig. 3A). Second, the conformation of loop 222–226 at the entrance of the solvent-exposed substrate pocket in the protomer differed between the two structures (Fig. 3A, B, Supplementary Fig. S5). Loop 222–226, known as the ATEYS loop, comprises five residues (Ala-Thr-Glu-Tyr-Ser) and is highly conserved in FAD-dependent oxidoreductases from *Pseudomonas* species. In the cryo-EM structure, the ATEYS loop was slightly distant from FAD, and the substrate pocket was wide open to the solvent (Fig. 3C). In contrast, the X-ray crystallographic structure indicated that the ATEYS loop was in a closed conformation and covered the substrate pocket. Both X-ray crystallography and cryo-EM structures showed that the ATEYS loop exhibited higher temperature factors than the surrounding area (Fig. 3D). These results suggest that the ATEYS loop is highly mobile and may play an important role as a lid at the entrance of the substrate pocket in solution.

### Substrate-binding pocket of PT-AROD

A previous study reported that PT-AROD exhibits high substrate specificity, with 100% activity towards L-arginine, only 9.9% activity towards L-lysine and negligible activity towards other amino acids *(**17**)*. To better understand the mechanism by which ARODs recognize substrates, we studied an ancestral lysine oxidase, namely AncLLysO (PDB ID: 7EIH), which had the highest sequence identity (32%) with PT-AROD amongst LAAOs with structures deposited in the PDB. AncLLysO is reported to have >30% relative activity towards L-arginine whilst showing the highest activity towards L-lysine at high substrate concentrations *(**41**)*. Comparative analysis of our PT-AROD structure by Cryo-EM and the AncLLysO structure revealed that the RMSD of Cα atoms between the monomers was 1.070 Å (Fig. 4). Furthermore, an activity-deficient mutant was used to determine the structures of AncLLysO with L-lysine and L-arginine as substrates (PDB ID: 7EII and 7EIJ, respectively) *(**41**)*. Superposition of the structures of AncLLysO (chain B in 7EIJ) and PT-AROD showed an RMSD of 1.042 Å for Cα (Fig. 4). Residues of AncLLysO involved in substrate binding were compared with those of PT-AROD. In AncLLysO, the side chain of Glu383 and the main chain of Gly553 interact with the side chain of the substrate L-arginine. In particular, the side chain of Glu383 also interacts with the side chain of the substrate L-lysine, suggesting that lysine oxidase requires Glu383 to recognize the side chains of substrates.

**Table 3:** The kinetic parameters of the wild-type PT-AROD and mutants for L-arginine and L-lysine

	L-arginine			L-lysine		
	Wild type	E493A	Q350E	Wild type	E493A	Q350E
*K*_m_ (μM)	4.8 × 10^2^	1.9 × 10^2^	1.7 × 10^3^	2.0 × 10^4^	1.2 × 10^3^	6.9 × 10^3^
*k*_cat_ (s^−1^)	6.2	3.5	10.4	2.7	1.1	0.5
*k*_cat_/*K*_m_ (s^−1^·M^−1^)	1.3 × 10^4^	1.8 × 10^4^	6.1 × 10^3^	1.4 × 10^2^	9.2 × 10^2^	7.2 × 10

**Table 4:** Substrate specificity of PT-AROD wild type and mutant for 20 types of amino acids

	Relative activity (%)	
	Wild type	E493A mutant
L-Arg	100	100
L-Lys	18	22
L-Phe	2	12
L-Leu	n.d.	10
L-Met	1	7
L-His	1	2
L-Asn	1	2
L-Pro	1	1
L-Ser	1	1
L-Ala	n.d.	n.d.
L-Asp	n.d.	n.d.
L-Cys	n.d.	n.d.
L-Gln	n.d.	n.d.
L-Glu	n.d.	n.d.
Gly	n.d.	n.d.
L-Ile	n.d.	n.d.
L-Thr	n.d.	n.d.
L-Trp	n.d.	n.d.
L-Tyr	n.d.	n.d.
L-Val	n.d.	n.d.

The response to 10 mM of each amino acid was measured at 37°C. The activity towards each amino acid is expressed relative to that towards L-arginine, which has an absorbance of 100%. n.d. means not detected. Amino acids are represented by their three-letter codes.

### Analysis of PT-AROD mutants

We first focused on Glu383 in AncLLysO, which corresponded to Gln350 in PT-AROD, to design the Q350E mutant of PT-AROD, which was expected to exhibit enhanced activity towards L-lysine. In addition, analysis of the binding sites of AncLLysO and PT-AROD suggested that Glu493 in PT-AROD was proximal to the substrate side chain (Fig. 4). This residue corresponded to Ala518 in AncLLysO, and thus Glu493 in PT-AROD might also contribute to its relatively high selectivity for L-arginine. Therefore, we evaluated the reactivity of the Q350E and E493A mutants that mimic AncLLysO towards L-arginine and L-lysine. Table 3 shows the enzymatic parameters of wild-type PT-AROD, Q350E and E493A mutants for the two substrates. A comparison of the kinetic constants(*k*_cat_/*K*_m_) revealed that the Q350E mutant exhibited lower values for L-arginine than the wild type and E493A. Furthermore, the Q350E mutant had a higher *K*_m_ for L-lysine than the wild type, yet its *k*_cat_ was ~19% lower, resulting in a *k*_cat_/*K*_m_ value that was ~49% lower than that of the wild type. It was previously demonstrated that Glu383 of AncLLysO interacted with the side chain of the substrate, suggesting that this residue is important for substrate recognition. However, the results of the Q350E mutant assay suggest that this mutation may be an obstacle to the substrate binding of PT-AROD. Conversely, the E493A mutant exhibited 1.5- and 6.1-fold higher *k*_cat_/*K*_m_ values for L-arginine and L-lysine, respectively, in comparison with the activity of the wild-type PT-AROD. The E493A mutation facilitates the entry of these amino acids into the substrate binding site. Thus, the reactivity of the E493A mutant to other standard amino acids was examined. The specific activities of the wild type and the E493A mutant for 10 mM L-arginine were 26.1 and 30.8 units/mg, respectively. When the specific activity for L-arginine was considered to be 100%, the E493A mutant showed higher reactivity for L-lysine, L-phenylalanine, L-leucine, L-methionine, L-histidine and L-asparagine (Table 4). These findings suggest that the Glu493 residue of PT-AROD plays a role in conferring high substrate specificity. In contrast, the activity of the E493A mutant towards L-lysine was ~22% of that towards arginine and did not show as high an activity towards L-lysine as AncLLysO. This finding suggests that not only the E493A mutation, but also other residues around the entrance of the substrate pocket and its binding site contribute to the functional divergence of lysine oxidase and arginine oxidase. These results offer significant insights into the substrate specificity of amino acid oxidase and provide a foundation for the engineering of enzymes that can distinguish between L-arginine and L-lysine in amino acid assays.

## Conclusion

We determined the structure of PT-AROD from *Pseudomonas* sp. TPU 7192 using cryo-EM at 2.3 Å resolution and X-ray crystallography at 3.4 Å resolution. In the cryo-EM structure, PT-AROD formed an octameric assembly with D4 symmetry. In addition, one molecule of FAD was bound to each protomer, and intermolecular disulphide bonds existed between Cys390 and Cys579. Comparative analysis of the structures determined using cryo-EM and X-ray crystallography revealed that the ATEYS loop located at the entrance of the substrate pocket functioned as a lid. Furthermore, comparative analysis of these structures and that of an ancestral lysine oxidase, which had the highest degree of sequence homology with PT-AROD amongst the proteins in the PDB, revealed that residue Glu493 was important for interactions of PT-AROD with substrates. The results of site-directed mutagenesis indicated that Glu493 contributed to the substrate specificity of PT-AROD. The structure obtained in this study represents the first example in the AROD family, and the present results provide important information about the substrate selectivity of amino acid oxidase for industrial applications.

## Supplementary Material

Web_Material_mvae070
