# Supplementary material for: Open and closed structures of L-arginine oxidase by cryo-electron microscopy and X-ray crystallography
Source: J Biochem. 2024 Oct 18;177(1):27–36. doi: 10.1093/jb/mvae070 (PMC11694665; doi:10.1093/jb/mvae070)
Supplement: Web_Material_mvae070 [file web_material_mvae070.zip › Supplementary caption.docx]

**Supplementary Figure 1 Molecular distribution by mass photometry (N = 3)**

The three molecular distribution results of PD-AROD are superimposed (N = 3). Each line shows the best-fit Gaussian distributions.

**Supplementary Figure 2 Density map of disulfide bond between Cys390 and Cys579 in molecular interface**

A disulfide bond was observed between Cys390 (left) and Cys579 (right) at the molecular interface of the oligomer.

**Supplementary Figure 3 Storage stability of PT-AROD in solution at 4℃**

Residual activity after incubation at 4°C is shown as relative activity with the initial value as 100%

**Supplementary Figure 4 PT-AROD structure using X-ray crystallography.**

Red box is a unit cell. Four molecules of PT-AROD in the asymmetric unit are shown in four colors and symmetry-related molecules are shown in gray.

**Supplementary Figure 5 Density maps of ATEYS loops in cryo-EM and X-ray crystallography structures.**

(left) The density map of the ATEYS loop in cryo-EM structures (counter level 0.031 using UCSF ChimeraX) (right) The electron density map of ATEYS loop in X-ray crystallography (sigma level 1.0 using pymol 3.0.2)
