# Supplementary figures and images for: Open and closed structures of L-arginine oxidase by cryo-electron microscopy and X-ray crystallography
Source: J Biochem. 2024 Oct 18;177(1):27–36. doi: 10.1093/jb/mvae070 (PMC11694665; doi:10.1093/jb/mvae070)

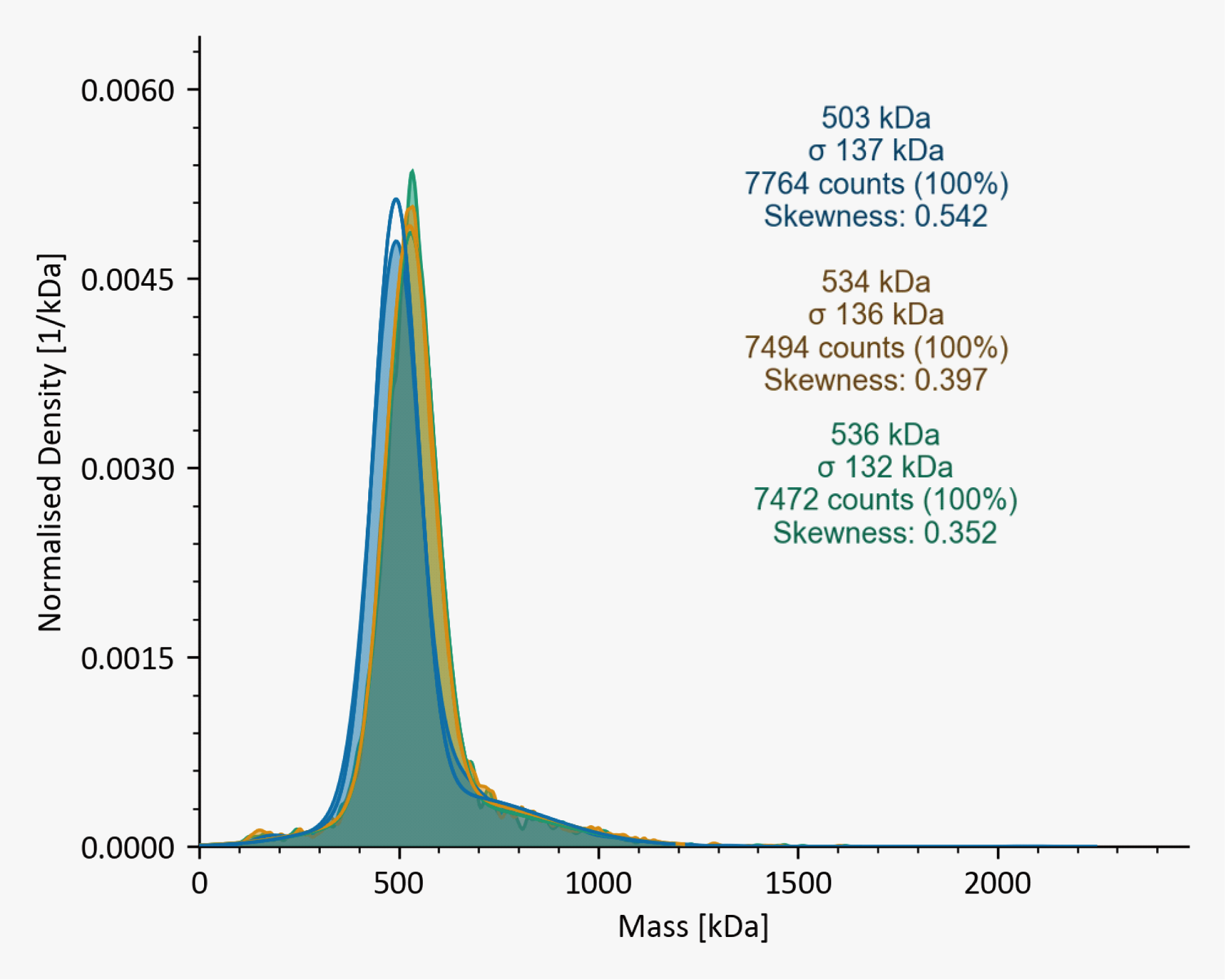

Supplement: Web_Material_mvae070 [file web_material_mvae070.zip › FigS1.tif]

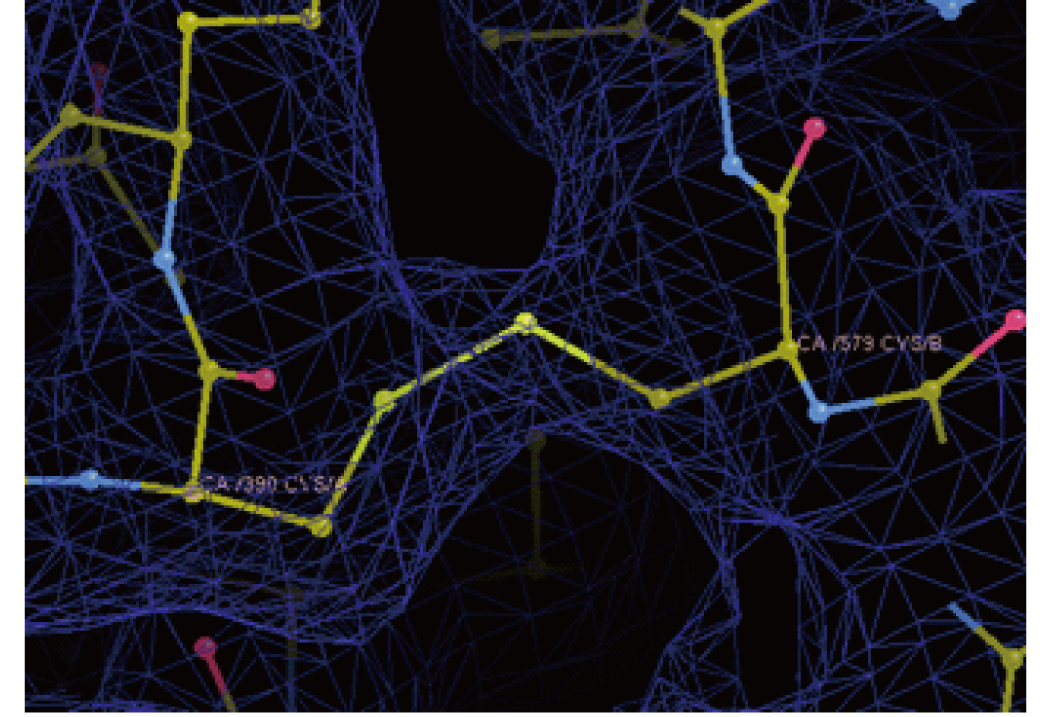

Supplement: Web_Material_mvae070 [file web_material_mvae070.zip › FigS2.tif]

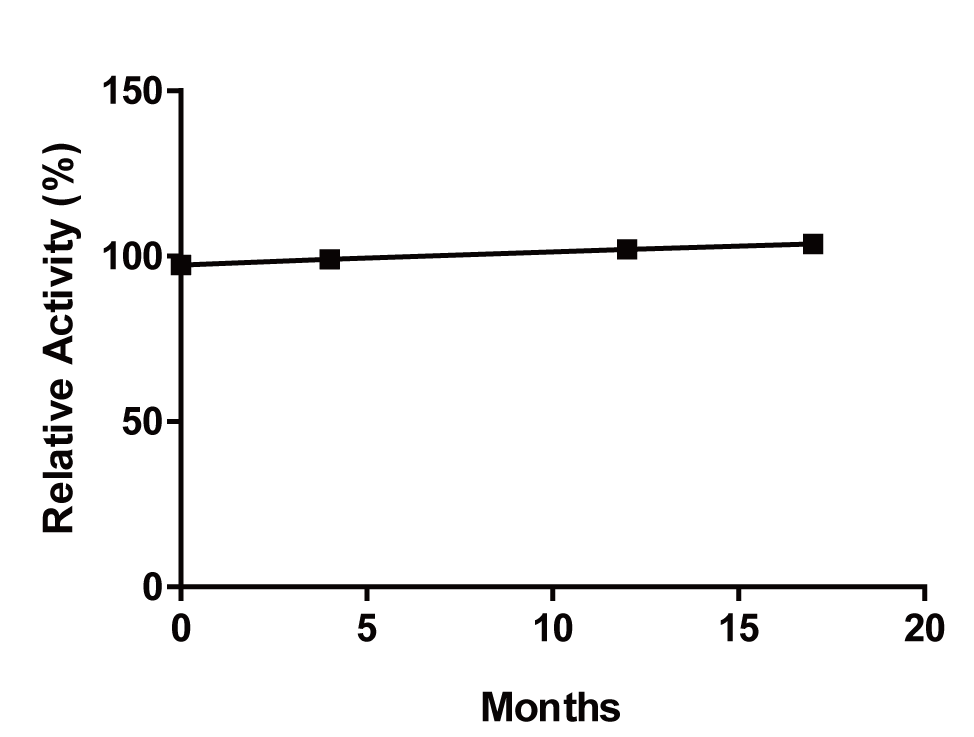

Supplement: Web_Material_mvae070 [file web_material_mvae070.zip › FigS3.tif]

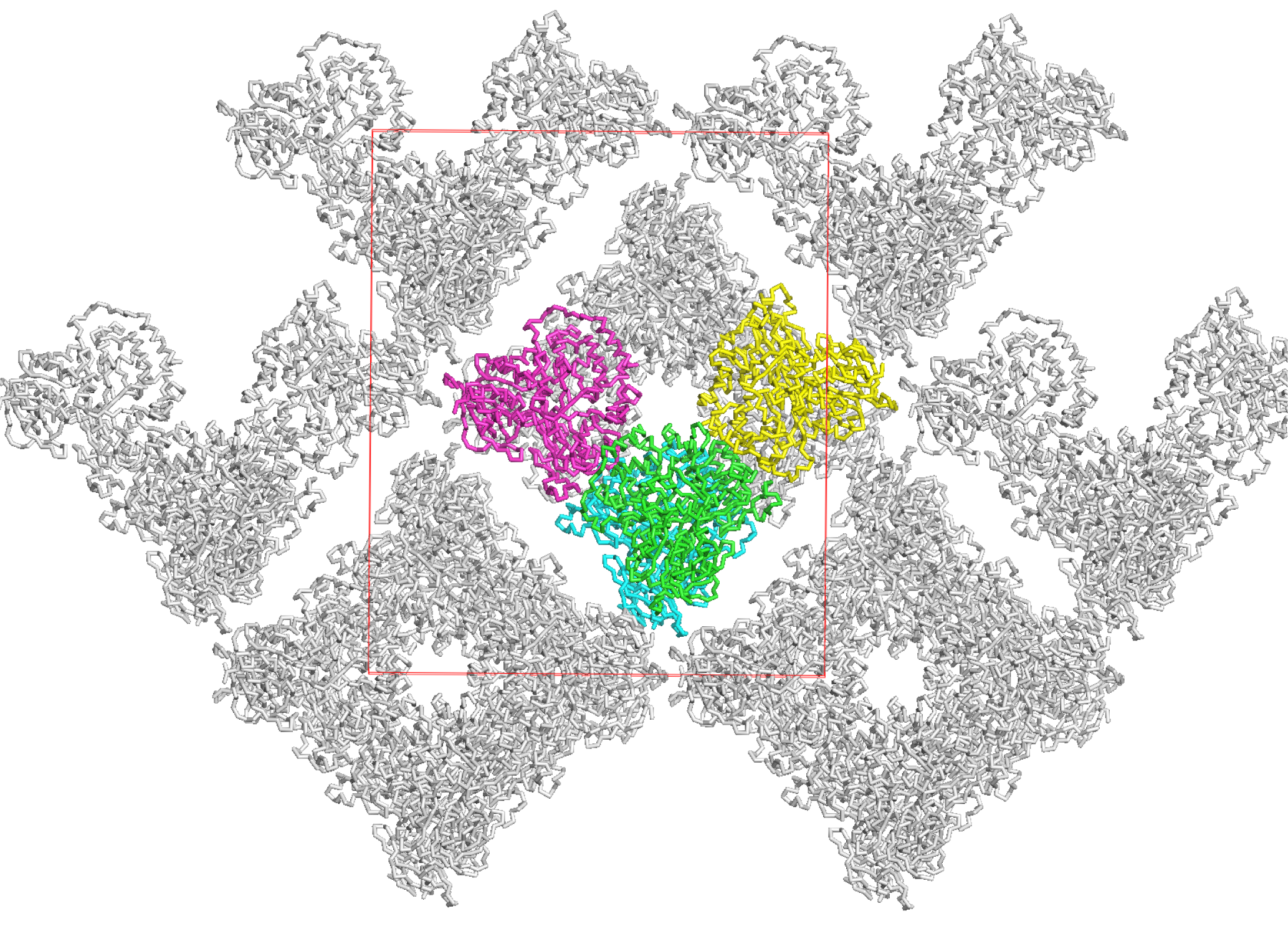

Supplement: Web_Material_mvae070 [file web_material_mvae070.zip › FigS4.tif]

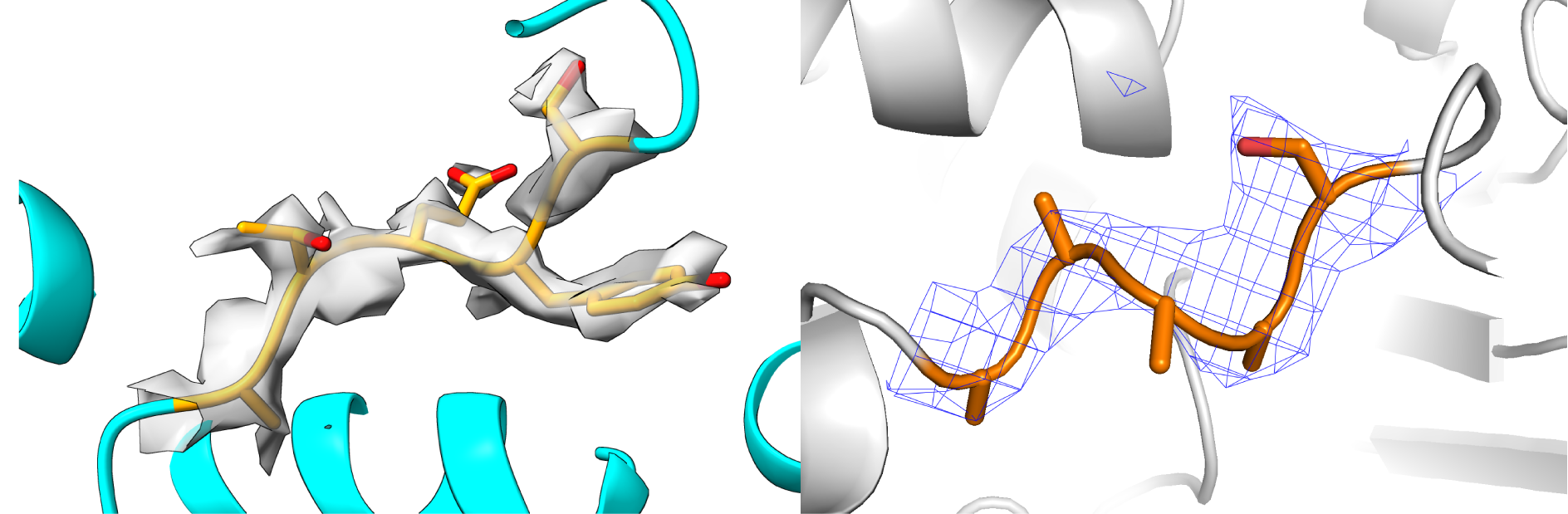

Supplement: Web_Material_mvae070 [file web_material_mvae070.zip › FigS5.tif]
